# Competing magnetic effects due to the incorporation of oxygen in thin films of (ZnCo)O

**DOI:** 10.1039/c9ra06899f

**Published:** 2019-11-21

**Authors:** Ahmad M. A. Saeedi, Fatma M. Gerriu, Minju Ying, Marzook S. Alshammari, Steve M. Heald, Xiaoli Li, Harry J. Blythe, A. Mark Fox, Gillian A. Gehring

**Affiliations:** Department of Physics and Astronomy, University of Sheffield Hicks Building S3 7RH UK g.gehring@shef.ac.uk; Department of Physics, Umm Al-Qura University P.O.Box 715 Makkah 21421 Saudi Arabia; Department of Physics, Misurata University Libya; Key Laboratory of Beam Technology of Ministry of Education, College of Nuclear Science and Technology, Beijing Normal University Beijing 100875 China; The National Centre for Laser and Optoelectronics, King Abdulaziz City for Science and Technology, KACST P.O. Box 6086 Riyadh 11442 Saudi Arabia; Advanced Photon Source, Argonne National Laboratory Argonne IL 60439 USA; Key Laboratory of Magnetic Molecules and Magnetic Information Materials of Ministry of Education, School of Chemistry and Materials Science, Shanxi Normal University Linfen 041004 People's Republic of China

## Abstract

We have investigated the magnetic properties of ZnCoO thin films grown by pulsed laser deposition from targets made from pure ZnO combined with metallic Co, CoO or Co_3_O_4_ as a function of oxygen pressure in the deposition chamber. We find that the structural and magnetic properties of films grown from targets containing CoO or Co_3_O_4_ are similar and can be mapped on to each other by assuming that the films made from CoO require some additional oxygen to make them the same as those grown from Co_3_O_4_. The data suggest that the magnetism in these films is due to oxygen vacancies. Radically different properties are seen for the films grown with metallic Co in the target. In this case, there is structural evidence for the production of Zn vacancies as oxygen was added during deposition and this was accompanied by a strong increase of the magnetisation. In contrast, there was very little difference seen between the magnetic properties of the targets, which were all found to be paramagnetic, even after further annealing in air.

## Abstract 2

The properties of laser ablated films of ZnCoO depend on the cobalt precursor used to make the target.

## Graphical abstract

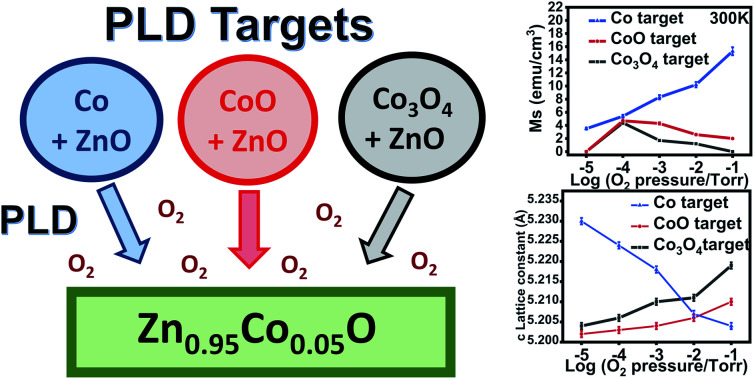

## 1. Introduction

Transition metal doped ZnO films, nanoparticles and nanowires have been studied intensively since the first theoretical predictions that they should be ferromagnetic if they were n-type^1^ and if they were p-type.^2^ ZnO is a promising material because it is readily available, is bio-compatible, is piezoelectric and has a wide direct bandgap. Hence there have been many proposed applications in spintronics, optoelectronics and medicine.^3,4^

There have been studies that show that the doping with transition metals is not essential; pure ZnO can support a considerable magnetism, provided it contains sufficient structural or stoichiometric defects; however, the magnetism is enhanced by the addition of transition metals. Grain boundaries are one such lattice defect and films of ZnO have been shown to be magnetic if the grain size is sufficiently small (less than ∼20 nm).^5^ Magnetism was also found in films, if they were sufficiently thin, so that surface states can support magnetism.^6^ Ion implantation introduces many lattice defects including vacancies and interstitials that are also effective in producing magnetism, as has been shown with implantation of As and Kr.^7,8^ There have also been studies where specific defects have been formed by growing the samples away from stoichiometry.

Many other oxides, In_2_O_3_, TiO_2_, SnO_2_, HfO_2_, are magnetic if grown oxygen deficient^9^ and this has also been shown to occur for the surface of a ZnO crystal.^10^ This magnetism is attributed to oxygen vacancies. However, ZnO is unusual in showing magnetic properties when it is grown oxygen-rich, as it was found that magnetism was present when the film was very thin (∼50 nm); however this would disappear after the film was annealed *in vacuo*.^11^ A film made by sol–gel techniques, which was rich in oxygen, also showed strong magnetism that was attributed to Zn vacancies.^12^ It is worth noting that these non-stoichiometric samples have surface effects due to being either very thin films or having small grains; thus, it has not always been easy to separate the effect of Zn or oxygen vacancies from the effects due to grain boundaries or surfaces.

The behaviour of the saturation magnetic moment of most of these defective oxides drops rather slowly, if at all, between low temperature and room temperature. This was first explained by Coey^13^ who observed that such behaviour occurs in Stoner magnets where the moment is entirely due to a polarised band and the Curie temperature, *T*_c_, which is related to the Fermi energy so is well above room temperature. This contrasts with materials where the magnetism is due to a local moment with spin *S* where the transition temperature is of the order of *U*/*k*_B_ log_e_(2*S* + 1) where *U* is the ground-state energy. Transition temperatures of dilute local moment magnets, for example GaMnAs, are typically below room temperature.

It appears that narrow defect bands associated with both donors and with acceptors can occur in ZnO with properties such that the Stoner criterion for spontaneous magnetism can be met. In most oxides, that are only magnetic when grown sufficiently oxygen poor, this means that the conditions can only be met for donor bands associated with oxygen vacancies. A donor band requires that coherent transport occurs between defect sites, which means that the wave functions of the electrons in the defect states should have a radius that is larger or comparable to the separation between defects. This requires a small effective mass and a high dielectric constant. However, the defect states must be sufficiently deep so that the defect bands do not overlap the conduction or the valence band, because this would lead to a wider band and hence a lower density of states. This combination of a relatively narrow band-gap and deep defect levels appears to occur for ZnO alone. It thus appears to be the only oxide in the ‘Goldilocks zone’ for d^0^ magnetism when it is both oxygen poor and oxygen rich.

There have been many studies of ZnO doped with transition metals. We consider particularly the results obtained with Co. One of the problems with ZnCoO is that metallic Co is strongly magnetic, so there is a challenge to separate intrinsic effects from those arising from Co nanoparticles; this is particularly critical if the films were grown oxygen-deficient as this makes the precipitation of Co nanoparticles more favourable.^14,15^ There have been conflicting theoretical predictions as ZnCoO has been predicted to be ferromagnetic if it is p-type^2,16^ and n-type if it is due to being oxygen deficient or doped with Al.^1,17^ There are many works that discuss the relevance of magnetic polarons based on Co^2+^ ions.^18^ However, there have been measurements using XMCD that have shown that the Co ions are paramagnetic even if the sample is ferromagnetic.^19^

In some cases it has been useful to correlate the behaviour of ZnCoO with that of pure ZnO; in these cases it is generally found that the effect of adding Co is to enhance the effect that was already present for pure ZnO, for example, it was shown that there was a critical grain size for ZnCoO to be magnetic, but that this occurred for larger grains than were required for pure ZnO.^20^ It was also found that high quality ZnCoO films were not magnetic, thus indicating that crystalline defects are playing a role here as in the case of pure ZnO.^21,22^

Films have been made by Pulsed Laser Deposition (PLD), reactive sputtering, annealed sol–gel and by ultrasonic assisted solution chemical vapour deposition.^23–26^ There have been studies of the effect of changing the carrier concentration on the magnetic properties, by co-doping with Al or Ga and by growing with various amounts of oxygen.^25,27–30^ The majority of samples have been grown such that they were n-type conductors and it was reported that the magnetism disappeared as the amount of oxygen in the films was increased, thus supporting the hypothesis that oxygen vacancies or Zn interstitials were necessary for the observation of room temperature magnetism.^26,27^ However, there have also been reports of substantial magnetism being observed when the films were grown oxygen-rich.^24,28^

Most of the authors who report work on films grown by PLD have used a ceramic target of ZnCoO that has been fabricated using solid state reaction techniques. In most cases the Co has been introduced by using Co_3_O_4_ as the precursor,^23,27^ however CoO and metallic Co have also been used.^31,32^ In this study, we compare the results of using three different precursors: Co metal, CoO and Co_3_O_4_, to make the PLD targets and also grow films at different oxygen pressures. This enables us to study to what extent adding oxygen to the target is equivalent to adding oxygen during ablation in the PLD chamber.

This paper is a longer and much more detailed account of these films than that which we had given in an earlier letter.^33^ There is also an important difference between the films studied. In the earlier letter the ablation was carried out for a given time that was the same for all targets and all oxygen pressures. As a result the films described in the APL were thick when grown at base pressure and much thinner when grown in an oxygen atmosphere. Hence, there was the possibility that some of the difference between the films grown at different oxygen pressures might have been due to the different film thicknesses.

In this work films, of similar thickness, were made from all three targets at all oxygen pressures. Hence results from the films made with the CoO and Co_3_O_4_ at different oxygen pressures are also included in detail. Magnetic studies of the targets are also included and this data is contrasted with that obtained for the films. Optical absorption and carrier concentration data are also included here. We show data that demonstrates that the effects of using different targets persist for all the values of oxygen pressure in the PLD chamber.

## 2. Experimental methods

Targets of the same nominal composition, Zn_0.95_Co_0.05_O, were prepared from high-purity ZnO powder combined with powders of metallic Co, CoO, or Co_3_O_4_. All the powders, which had purities of 99.999% for ZnO, 99.998% for metallic Co 99.998% for CoO, and 99.998% for Co_3_O_4_, were purchased from Alfa Aesar. In each case, the process of first grinding and then sintering the mixture in air for 12 hours was repeated for annealing temperatures of 400 °C, 600 °C, and 800 °C, before finally pressing into a target mould and sintering at 1000 °C for more than 12 hours.^33^ The targets all appeared similar and were green in colour, which indicates that similar amounts of the Co had been incorporated into the ZnO lattice during the solid state reaction to form the compound Zn_1−*x*_Co_*x*_O, which is known as cobalt green (or Rinmann's green). In what follows, the ablated films will be referred to by the precursor used in the target's preparation, *i.e.* as Co, CoO and Co_3_O_4_ films.

A Lambda Physik LEXTRA 200 XeCl excimer laser with an operating wavelength of 308 nm and using a 10 Hz repetition rate was used for the ablation of the target. The separation between the target and the substrate was *ca.* 3.5 cm. All the films were deposited on double-sided, polished, sapphire substrates that had been heated to 450 °C. The substrates had been purchased from PI-KEM Ltd and were single-crystal *c*-cut Al_2_O_3_. Five films were grown from each target: one at base pressure of 2 × 10^−5^ torr and four at higher oxygen pressures of 1 × 10^−4^, 1 × 10^−3^, 1 × 10^−2^ and 1 × 10^−1^ torr. The deposition times were varied so that the thicknesses of all the films were comparable and lay between 100 nm and 130 nm.

The lattice constants of the films and the grain sizes were measured using X-ray diffraction using CuKα radiation (*λ* = 1.5406 Å) and the chemical state and physical environment of the Co ions were studied using X-ray absorption techniques, XANES and EXAFS.

The magnetic properties of both the films and the targets have been studied using a SQUID magnetometer. The magnetisations of the films were measured with the applied magnetic field in the plane of the sample. Measurements were made of the magnetisation as a function of temperature in field-cooled (FC) and zero-field-cooled (ZFC) conditions. Hysteresis loops were also measured at room temperature and at low temperatures. The magnetisation of the sapphire substrate was also measured and this contribution was subtracted from the measurements of all the samples shown here.

The nature of the states located in the energy gap of ZnO were investigated using optical absorption and magnetic circular dichroism (MCD) which is measured in Faraday geometry in the presence of a magnetic field parallel to the direction of light propagation. The MCD signal from a film of thickness *L* is defined as the difference in absorption of right and left circularly polarised light. This is a powerful technique because it is sensitive to the spin polarisation in either, or both, the initial and final states that are involved in the transition.^32,34,35^ The carrier concentration of the films was measured using a Quantum Design Physical Property Measurement System, PPMS.

## 3. Structural and chemical characterisation of the films

**Fig. 1:**
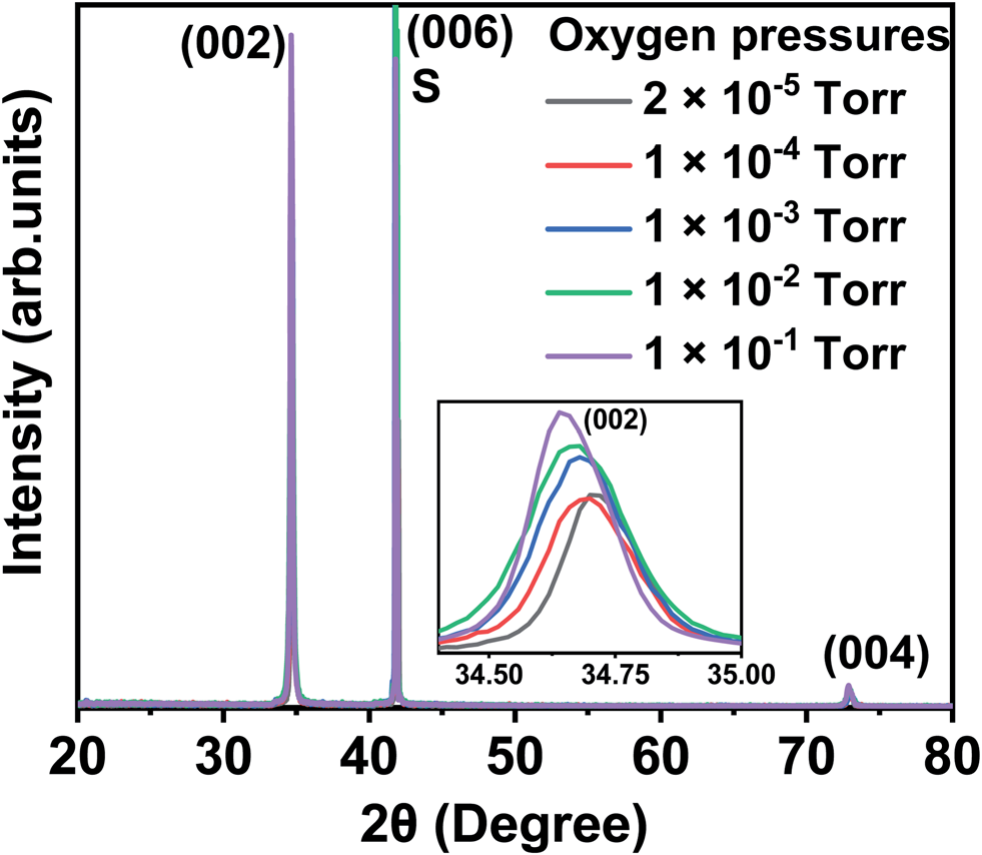
XRD data of the thin films grown from the Co_3_O_4_ target at a base pressure of 2 × 10^−5^ torr, and oxygen pressures of 1 × 10^−4^, 1 × 10^−3^, 1 × 10^−2^, and 1 × 10^−1^ torr. The inset demonstrates the shifting of the (002) peak towards smaller 2*θ* with increasing oxygen content in the chamber. S indicates the sapphire (006) peak.

**Fig. 2:**
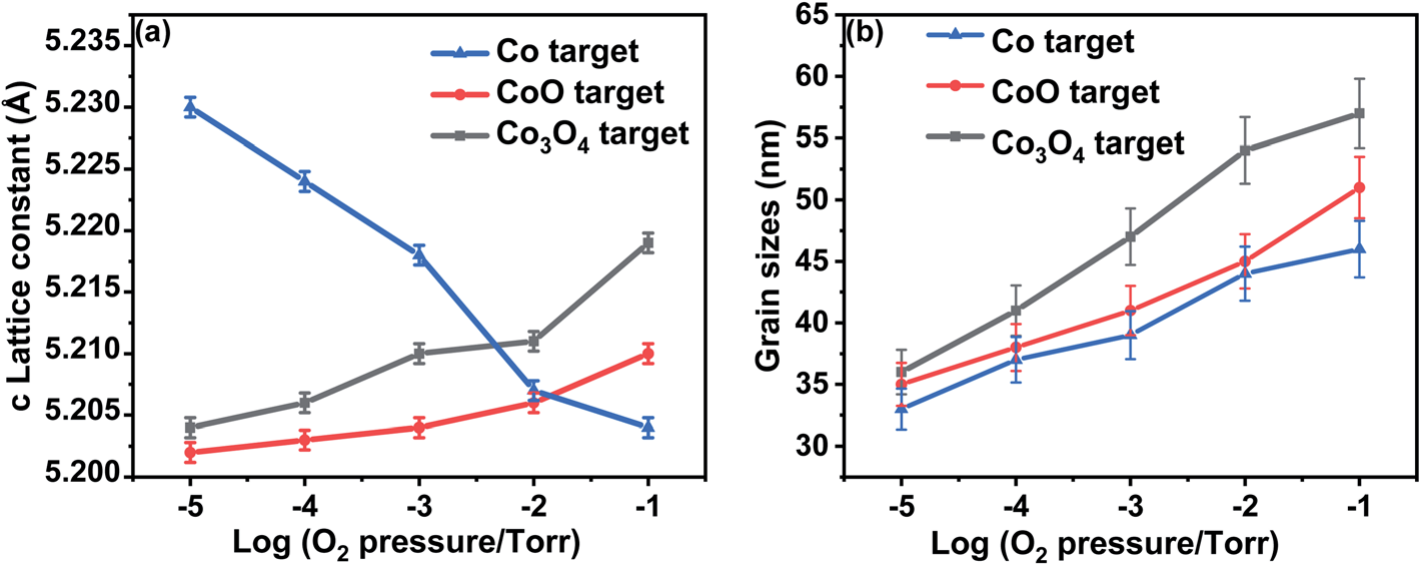
Lattice constants and the grain sizes extracted from the XRD data for the films ablated from targets made from Co, CoO and Co_3_O_4_ as a function of oxygen pressure (a) the *c* lattice constant for all films as a function of oxygen pressure. (b) The grain sizes for all the films grown from targets, as a function of oxygen pressure.

Typical XRD plots for films made from Co_3_O_4_ target grown at different oxygen pressures are shown in Fig. 1. A comparison of the data from all three targets is shown in Fig. 2. All the films exhibit a single phase with a typical wurtzite ZnO structure, showing a *c*-axis out of plane orientation with only (002) and (004) ZnO diffraction peaks observed. The (006) diffraction peak is from the sapphire substrate. No secondary phases were detected within the detection limit of the XRD for any of the films. The lattice constant is expected to contract as the concentration of vacancies rises and, conversely, to expand with increasing numbers of interstitials.^33^

The lattice constant increases as oxygen is added to the PLD chamber for the films made from CoO and Co_3_O_4_ targets; this is consistent with the films having a large number of oxygen vacancies when grown at low oxygen pressure and that these vacancies are removed at higher oxygen pressure causing the lattice to expand. The lattice constants shown in Fig. 2(a) imply that the extra oxygen that is present due to using a Co_3_O_4_ target compared with CoO is very roughly equivalent to increasing the oxygen pressure in the PLD chamber by a factor of 10. This behaviour, namely that the effect of using different targets could be off-set by changing the oxygen pressure in the PLD chamber, was also seen for films of (InFe)_2_O_3_.^36^

Very different behaviour is observed for the film made from metallic Co as the lattice constant decreased when the oxygen pressure was increased, as is seen in Fig. 2(a). This effect was observed previously and was attributed to an increasing concentration of Zn vacancies with increasing oxygen in the PLD chamber.^33^

The enlarged ZnO (002) diffraction peaks, as shown in Fig. 1, were used to estimate the grain sizes using the Scherrer equation. The ranges of the grain sizes were found to be between 33 nm and 57 nm, which are in the acceptable range where magnetism might occur as predicted from this mechanism.^20^ The grain sizes increased with the amount of oxygen pressure in the chamber for all the films, as shown in Fig. 2(b). The largest grains were obtained for the films made from the Co_3_O_4_ target.

A more accurate estimate of the presence of defect phases, particularly to identify metallic Co and the precursors CoO and Co_3_O_4_ was obtained from an analysis of K-edge XANES and EXAFS spectra. The measurements were made using beamline 20-BM at the Advanced Photon Source. A Si (111) monochromator was used providing 1 eV energy resolution at the Co K edge. The measurements were made at room temperature at a glancing angle of ∼5° with the X-ray polarization normal to the surface of the films. Typically, 4–8 scans were averaged for an improved signal-to-noise ratio and the data were analysed using the *Demeter* analysis package.^37^

**Fig. 3:**
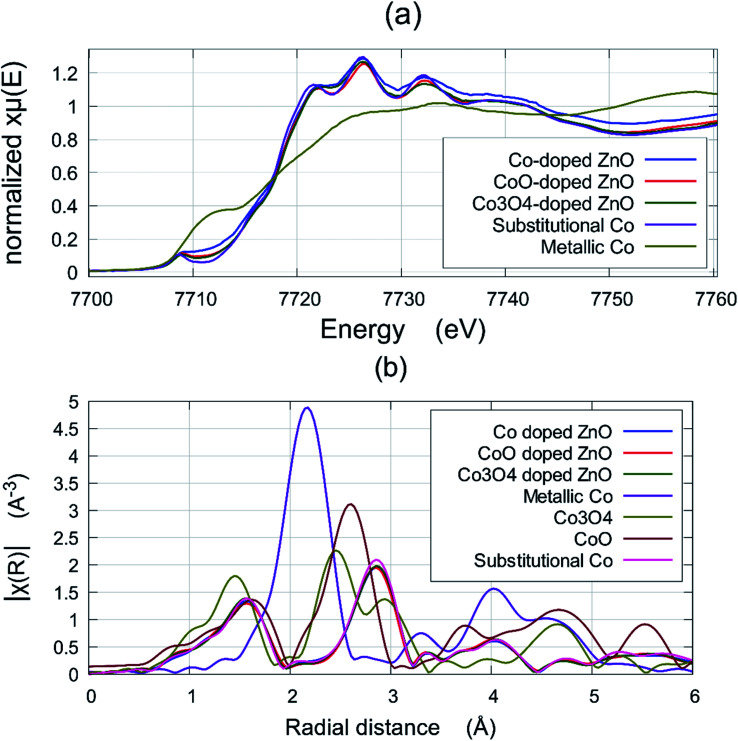
(a) The EXAFS signal of the films grown from Co, CoO and Co_3_O_4_ targets grown at base pressure compared with a reference spectrum of ZnCoO which contained all the Co on Zn sites and the spectrum of metallic Co; (b) the Fourier transform of the EXAFS of the films from Co, CoO and Co_3_O_4_ targets grown in 10^−3^ torr and the reference signal (these all superpose) compared with the signals from metallic Co bulk CoO and Co_3_O_4_ which are quite distinct.

This measurement can detect the existence of metallic Co and any secondary oxide phases. Fig. 3(a) shows a comparison of XANES data of the film deposited at a base pressure of 1 × 10^−5^ torr, and the standard valence states of pure metallic Co and a sample of ZnCoO which is known to be fully Co substitutional in the Zn^2+^ sites. The near edge region ∼7712 eV is most sensitive to the metal and an examination of this region shows clearly that all Co-doped ZnO films that were deposited at base pressure from all three targets contain some metallic Co. The fraction of Co ions found to be in a metallic environment was estimated using the *Athena* and *Artemis* interface program packages,^14,37^ indicating that a percentage of such ions to be ∼28%, 16%, and 14% for Co-doped ZnO thin films grown at base pressure from the Co, CoO, and Co_3_O_4_ targets, respectively. No sign of metallic Co was seen in any of the films that were grown in an oxygen pressure of 10^−4^ torr, or any higher oxygen pressure.

It was important to ascertain if either of the oxide precursors, CoO or Co_3_O_4_, were present in the films and this was done best with EXAFS. Fig. 3(b) shows the Fourier transform of the EXAFS in order to compare the signals from the films grown at 10^−3^ torr with a film where the Co was known to be substitutional (these plots actually all superpose) with the signals from metallic Co, CoO and Co_3_O_4_ are shown in comparison. This figure demonstrates that any residual concentration of CoO or Co_3_O_4_ is below the detection limit.

The films grown from the target made with metallic Co contain the most metallic Co when grown at base pressure, which is not surprising. It was significant that none of the films grown at higher oxygen pressures showed any evidence for the presence of the oxides CoO or Co_3_O_4_. The variation of the lattice constant with pressure is the most revealing of the structural measurements as it shows that the films grown from a target formed from metallic Co are significantly different from those grown from a CoO or Co_3_O_4_ target.

## 4. Magnetic studies

The magnetic properties of both the films and the targets have been measured using the procedures described in Section 2.

### (A) Measurements of FC/ZFC magnetisation

#### (a) Targets

**Fig. 4:**
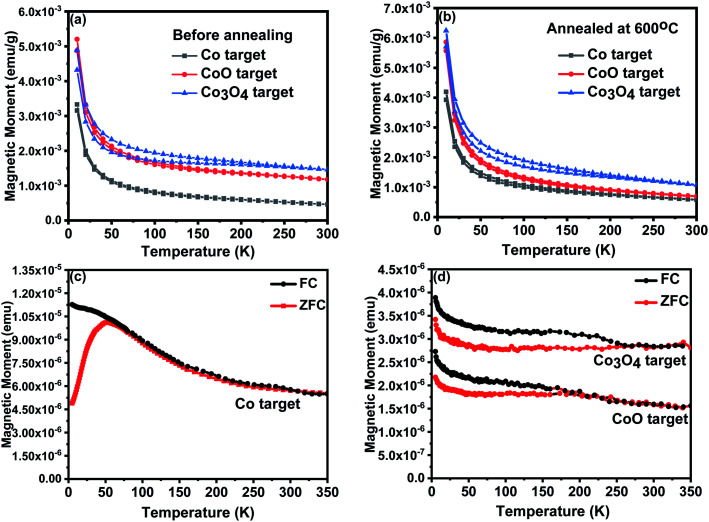
FC/ZFC magnetisation plots in an applied field of 100 Oe for the targets (a) as grown and (b) after a further anneal and of the films grown at base pressure of 2 × 10^−5^ torr for (c) the film grown from the Co target (d) films grown from the CoO and Co_3_O_4_ targets.

These measurements, made in a magnetic field of 100 Oe, were performed for the three targets, both as used for the deposition and after annealing in air; the results are shown in Fig. 4(a and b). Surprisingly, the only target to show hysteretic behaviour was the one made with Co_3_O_4_; this may be because of nano-particles of CoO which have been found to show weak ferromagnetism at room temperature.^38^ The lowest susceptibility was found with films prepared from the Co target and this showed no evidence of Co nanoparticles. Powders from the targets which were annealed in air at 600 °C for 1 hour were studied in order to investigate if similar behaviour occurred for the targets as was seen for films ablated in an oxygen atmosphere. However relatively little difference was seen after the anneal as shown in Fig. 4(a and b) and the effective moments are given in Table 1.

**Table 1:** The *p*_eff_ for all the targets before and after annealing at 600 °C for 1 hour

Samples	*p*_eff_		
	Co target	CoO target	Co_3_O_4_ target
Before annealed	0.8	1.1	0.9
Annealed at 600 °C	1	1.4	1.3

#### (b) Films

The FC/ZFC plots for the films grown at base pressure are shown in Fig. 4(c and d). There is a clear ZFC peak around 55 K for the film that was made with metallic Co as the precursor, which is also close to where the irreversibility sets in. This behaviour is that which would be expected for a film containing Co nanoparticles that are superparamagnetic above 55 K and become blocked below this temperature.^32^ The size of the nanoparticles is deduced to be ∼5.1 nm, when we used the magnetic anisotropy constant for bulk Co metal, *K* ∼ 2.7 × 10^5^ J m^−3^. This can be compared with the work of Ying *et al.*^33^ who did not find clear evidence for such nanoparticles in thicker films, 730 nm, grown under the same conditions from a Co target. The XANES data showed that the films made from the CoO and Co_3_O_4_ targets at base pressure contain some metallic Co, 16% and 14% respectively, but the magnetisation plots shown in Fig. 4(d) are clearly much smaller than those for the film grown from the Co target and any blocking is less clear, although they certainly show hysteretic behaviour at low temperatures. Interestingly the hysteretic behaviour for the film grown from the Co_3_O_4_ target showed an onset to hysteretic behaviour at a similar temperature as was seen for this target.

### (B) Hysteresis loops from films and targets

#### (a) Targets

**Fig. 5:**
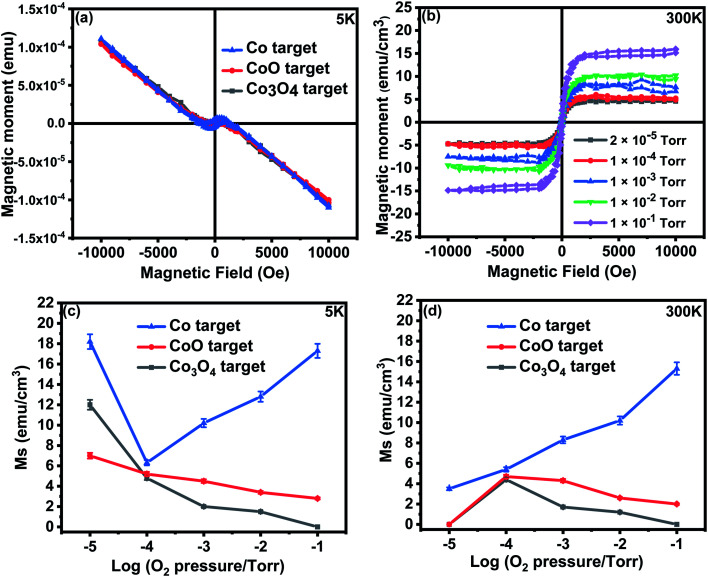
(a) Raw magnetisation measured at 5 K as a function of field for all films grown at base pressure (b) magnetisation measured at 300 K for the films grown from the Co target at different oxygen pressures, (c) saturation magnetisation as a function of O_2_ pressure measured at 5 K for all films (d) saturation magnetisation as a function of O_2_ pressure measured at 300 K for all films.

Hysteresis loops of small quantities of powders taken from the targets showed paramagnetic behaviour without any evidence for ordered magnetism for any of the targets down to 10 K. A comparison of the susceptibilities measured at 10 K and 300 K indicated that the data could be fitted to a Curie-Weiss law, *χ* = *C*/(*T* + *θ*) indicating antiferromagnetic interactions. The value of *θ* was found to be ∼10 K for all the targets. The value of the effective magnetic moment, *p*_eff_, was found for all the targets from the Curie constants. The values of *p*_eff_, all increased when the powders from the targets were annealed in air, which contrasts strongly with the magnetism observed for different values of films ablated at different oxygen pressures shown in Fig. 5(d).

#### (b) Films

Magnetic hysteresis loops measurements at 5 K and 300 K were taken for the films that were deposited at base pressure, 2 × 10^−5^ torr, and the four higher oxygen pressures 1 × 10^−4^, 1 × 10^−3^, 1 × 10^−2^, and 1 × 10^−1^ torr. The magnetic data for the films shown in Fig. 5(a) is the raw data before the substrate contribution was subtracted. The data, after subtraction of the contribution from the substrate, was separated into paramagnetic and ferromagnetic component. The ferromagnetic components for the films grown from the Co target are shown in Fig. 5(b) and the ferromagnetic components for all films are shown, as a function of O_2_ pressure, for 5 K and 300 K in Fig. 5(c and d).

The paramagnetic contributions found at 5 K and 300 K indicated Curie law behaviour. The Curie constant was used to evaluate the value of the effective moment, *p*_eff_ ≃ 3.4 ± 0.3 for all the films. This is close to the value of 3.8 expected for free spins of Co^2+^ and is in agreement with the work of Tietze *et al.*^19^ who showed that the Co^2+^ ions are all paramagnetic, even in ferromagnetic samples. This is much larger than the value of *p*_eff_ for all targets that was found to be 1.1 ± 0.3; this difference is probably due to antiferromagnetic interactions between Co ions in the targets.

The saturation magnetisation of the samples deposited at base pressure was strongly temperature dependent because of the existence of small nanoparticles that contributed to the magnetisation in a field of 1 T at 5 K, but not at 300 K. The saturation magnetism for the films grown in oxygen showed little temperature dependence, *M* (300 K)/*M* (5 K) ≥ 0.8, for all the samples indicating that donor band states rather than local moments may be involved.^13^

A comparison between the magnetic data for the films grown from the CoO and Co_3_O_4_ targets shows that the extra oxygen in the target made from Co_3_O_4_, compared with those made from CoO, is adding to the effects of the oxygen in the PLD chamber in determining the magnetisation of the film. The grain size increased with oxygen pressure, as shown in Fig. 2(b), so the reduction in the magnetisation of the samples made with an oxide precursor might be due to the reduction in the volume of the sample in grain boundaries, as well as the reduction of the density of oxygen vacancies. The magnetism of the sample grown with Co as the precursor was dramatically different from the others, indicating a different origin.

## 5. Optical and magneto-optical properties of the films

**Fig. 6:**
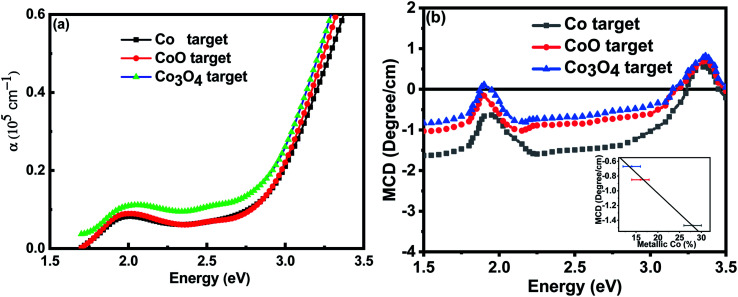
(a) The optical absorption of the films grown at base pressure showing considerable absorption below the energy gap due to defect states and the metallic Co nanoparticles. (b) The MCD of the samples; the inset shows a plot of the magnitude of the MCD at 2.5 eV plotted as a function of the fraction of the Co atoms in a metallic environment as estimated by XANES.

Full and empty energy bands do not contribute to the magnetisation so the observed magnetic moments must be due to polarised defect states lying in the band gap of 3.4 eV. The absorption spectrum and the MCD for the films grown at base pressure are shown in Fig. 6; the results focus on the region below the band gap at 3.4 eV because the samples were opaque at higher energies. The absorption shows that there is considerable absorption in the energy gap for all the films. This would be due to any metallic Co nanoparticles and also defect states possibly associated with the grain boundaries as the grain sizes were small ∼30 nm. There was significantly more absorption for the film made with the Co_3_O_4_ in spite of the fact that it contained the smallest percentage of metallic Co and also had the largest grains.

The MCD shows the expected dispersive signal between 1.9 eV and 2.1 eV which is characteristic of the d to d* transition for Co^2+^ ions^34^ which is superimposed on the broad negative signal from metallic Co embedded in ZnO.^32,39^ (The MCD signal from metallic Co nanoparticles is very strong so that it dominates the spectrum even where there are other, larger, contributions to the total magnetisation). The negative signal dominates the spectrum at 2.5 eV so the magnitude of this signal is plotted in the inset to Fig. 6(b) as a function of the fraction of the Co ions that were found to be in a metallic environment by XANES. The observed linear dependence confirms that this signal from the metallic Co is dominant as expected.

**Fig. 7:**
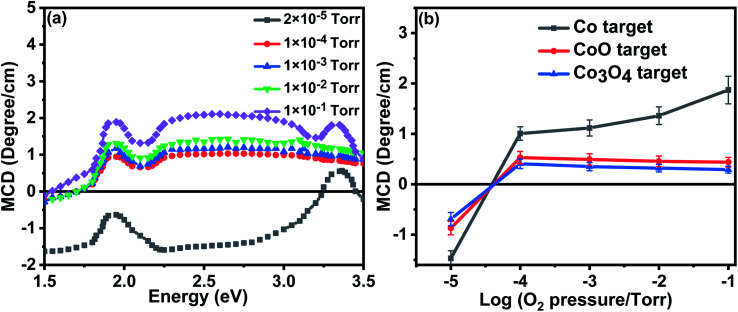
(a) The MCD spectra for the samples grown with the Co precursor for different amounts of oxygen in the PLD chamber (b) the MCD signal intensity at 2.5 eV for all the samples plotted as a function of oxygen pressure, the negative signal from metallic Co is only seen at base pressure.

The MCD spectra from the films that were grown in an oxygen-rich environment are very different; this is because they did not include a signal from metallic Co. Fig. 7(a) shows the MCD spectrum for the films grown with metallic Co as the precursor. Previous work on the MCD, in which the sample had a spin-polarised donor band, had given a negative signal.^35^ The positive sign observed here indicates that the transition is from a partially occupied spin polarised *acceptor* band to unoccupied band states.

Optical transitions occur between magnetically quantized electronic states with Δ*m*_s_ = 0 and Δ*m*_l_ = ±1 in Faraday geometry. Thus an MCD signal can only be observed if the spin and orbital states are mixed by spin–orbit coupling. In a partially filled orbital with positive spin polarisation, the induced orbital polarisation will be negative. This means that for transitions where the initial state is a polarised acceptor band and the final state is in the (unpolarised) conduction band, the intensity of the *σ*^+^ transition will be higher than that for *σ*^−^ leading to a positive MCD. The magnitude of the MCD at 2.5 eV for all the films is shown in Fig. 7(b). There is a similarity between the dependence of the room temperature magnetisation on oxygen pressure at values greater than 10^−4^ torr measured as shown in Fig. 5(d) and the MCD shown in Fig. 7(b).

## 6. Discussion

The results obtained here demonstrate that, for the targets CoO and Co_3_O_4_, the effects of having extra oxygen from the Co_3_O_4_ precursor has added to the effects of adding oxygen in the PLD chamber, as might have been expected from similar studies on In_2_O_3_.^36^ The effect was seen clearly in measurements of both the lattice constant and the saturation magnetisation. The effect of adding oxygen has been to reduce the fraction of oxygen vacancies and it was this that caused the lattice constant to increase and the magnetisation to decrease. It seems surprising that the effects of the precursor in the targets should be so important, even though the constituent powders were ground and sintered in air prior to being compressed into targets.

The results obtained when the precursor was metallic Co were dramatically different; in agreement work earlier work.^33^ This was in spite of the fact that there were very little significant differences in the properties of the targets. Hall effect measurements have shown that all the films, apart from one grown in an oxygen pressure in the PLD chamber, had densities of ∼10^18^ to 10^19^ n-type carriers per cm^3^ this was in agreement with the earlier deduction that a carrier density less than 10^19^ per cm^3^ was favourable for magnetism.^29^ (The exception was the film grown at the highest oxygen pressure, 100 mTorr, where no reliable data could be obtained). This agreed with the earlier work where a film of ZnO grown heavily oxygenated still had sufficient oxygen vacancies to give n-type carriers even though Zn vacancies were present.^12^ DFT calculations have shown that magnetism in undoped ZnO would be due to Zn rather than oxygen vacancies.^12,40^ However previous theory for Co doped ZnO has indicated that magnetism occurs due to oxygen vacancies as was seen here for the samples made from the CoO and Co_3_O_4_ targets.^41^ The experimental results included here show that the theories of Zn vacancy magnetism may also be applicable when Co^2+^ ions are present.

## 7. Conclusion

The measurements taken here indicate some of the subtleties of the PLD process. The physical properties and the magnetisation of the films depended on the precursor Co material used with ZnO to make the target. This occurred in spite of the fact that there was no evidence for either of the oxide precursors, CoO and Co_3_O_4_, in the films and no evidence of metallic Co in any of the films, including the one made with metallic Co in the target, provided that they were ablated in an oxygen pressure of 10^−4^ torr or higher. In addition, the targets themselves had similar magnetic properties with no obvious relation to those of the films made from them. The magnetic susceptibility of the powders, taken from the bulk targets, that were annealed at 600 °C for 1 hour in air, increased significantly. This must have been a surface effect since the whole target had previously been sintered at 1000 °C for 12 hours during fabrication. However, the effect of the exposure of the target powders to air during the anneal did not affect the target material in a way that bore any relation to the effects of adding small quantities of oxygen to the PLD chamber.

Any intrinsic magnetism that is observed in ZnCoO occurs due to the presence of Co ions and defects. Native defects that are discussed most widely in the context of magnetism are O and Zn vacancies. Their properties have been studied extensively.^42^

In this work, we have shown that the defects that occur for oxygen-poor and oxygen-rich samples can both give rise to magnetic properties for ZnCoO but that the type of defect magnetism observed depended on the precursor used to fabricate the target. In both cases, there is some evidence supporting the view that the magnetisation is due to correlated electrons in narrow defect bands rather than local moments associated with the Co or other defects. Furthermore, the type of defect in the films depends in a subtle way on the preparation of the target used for the PLD. A possible reason for the different behaviour of the films is that it is more likely that Co–O ion pairs are present in the laser-plumes of targets made from the oxides CoO and Co_3_O_4_, rather than for the films ablated from the Co target, where single ions of Co, Zn and O predominate.

The anomalous magnetic and structural behaviour seen for a target made from metallic Co and ZnO was all the more unusual as such behaviour did not occur when metallic Fe was used as the target with In_2_O_3_.^36^ We hypothesise that this is related to the fact that pure ZnO is unique among the transition metal oxides in becoming magnetic when it is grown strongly either oxygen-rich or oxygen-poor. This work offers new challenges for the understanding of the way that films grow following ablation from targets that differ in subtle respects.

Our work shows that there are considerable advantages in using metallic Co as the precursor in targets to be used for the fabrication of ZnCoO films by PLD. The role of the Co precursor is to facilitate the production of a narrow defect band based on Zn vacancies in films that were ablated in an oxygen atmosphere.

## Conflicts of interest

The authors have no conflict of interest.

## Supplementary Material

## References

[cit1] Sato K., Katayama-Yoshida H. (2001). Jpn. J. Appl. Phys., Part 1.

[cit2] Dietl T., Ohno H., Matsukura F., Cibert J., Ferrand e. D. (2000). science.

[cit3] Pan F., Song C., Liu X., Yang Y., Zeng F. (2008). Mater. Sci. Eng., R.

[cit4] Kim S., Cianfrone J., Sadik P., Kim K.-W., Ivill M., Norton D. (2010). J. Appl. Phys..

[cit5] Straumal B. B., Myatiev A., Straumal P., Mazilkin A., Protasova S., Goering E., Baretzky B. (2010). JETP Lett..

[cit6] Kapilashrami M., Xu J., Ström V., Rao K. V., Belova L. (2009). Appl. Phys. Lett..

[cit7] Ying M., Cheng W., Wang X., Liao B., Zhang X., Mei Z., Du X., Heald S. M., Blythe H. J., Fox A. M., Gehring G. A. (2015). Mater. Lett..

[cit8] Ying M., Saeedi A. M., Yuan M., Zhang X., Liao B., Zhang X., Mei Z., Du X., Heald S. M., Fox A. M., Gehring G. A. (2019). J. Mater. Chem. C.

[cit9] Hong N. H., Sakai J., Ruyter A., Brizé V. (2006). Appl. Phys. Lett..

[cit10] Hong N. H., Sakai J., Brizé V. (2007). J. Phys.: Condens. Matter.

[cit11] Zhan P., Xie Z., Li Z., Wang W., Zhang Z., Li Z., Cheng G., Zhang P., Wang B., Cao X. (2013). Appl. Phys. Lett..

[cit12] Xing G., Lu Y., Tian Y., Yi J., Lim C., Li Y., Li G., Wang D., Yao B., Ding J. (2011). et al.. AIP Adv..

[cit13] Coey J. M. D., Stamenov P., Gunning R., Venkatesan M., Paul K. (2010). New J. Phys..

[cit14] Heald S. M., Kaspar T., Droubay T., Shutthanandan V., Chambers S., Mokhtari A., Behan A. J., Blythe H. J., Neal J. R., Fox A. M., Gehring G. A. (2009). Phys. Rev. B: Condens. Matter Mater. Phys..

[cit15] Kaspar T. C., Droubay T., Heald S. M., Engelhard M. H., Nachimuthu P., Chambers S. A. (2008). Phys. Rev. B: Condens. Matter Mater. Phys..

[cit16] Spaldin N. A. (2004). Phys. Rev. B: Condens. Matter Mater. Phys..

[cit17] Qi S., Jiang F., Fan J., Wu H., Zhang S., Gehring G. A., Zhang Z., Xu X. (2011). Phys. Rev. B: Condens. Matter Mater. Phys..

[cit18] Coey J., Venkatesan M., Fitzgerald C. (2005). Nat. Mater..

[cit19] Tietze T., Gacic M., Schütz G., Jakob G., Brück S., Goering E. (2008). New J. Phys..

[cit20] Straumal B. B., Mazilkin A. A., Protasova S. G., Straumal P. B., Myatiev A. A., Schütz G., Goering E. J., Tietze T., Baretzky B. (2013). Philos. Mag..

[cit21] Tuan A. C., Bryan J. D., Pakhomov A., Shutthanandan V., Thevuthasan S., McCready D. E., Gaspar D., Engelhard M. H., Rogers Jr J., Krishnan K., Gamelin D. R., Chambers S. A. (2004). Phys. Rev. B: Condens. Matter Mater. Phys..

[cit22] Ney A., Ney V., Ye S., Ollefs K., Kammermeier T., Kaspar T., Chambers S., Wilhelm F., Rogalev A. (2010). Phys. Rev. B: Condens. Matter Mater. Phys..

[cit23] Ivill M., Pearton S., Rawal S., Leu L., Sadik P., Das R., Hebard A., Chisholm M., Budai J. D., Norton D. P. (2008). New J. Phys..

[cit24] Song C., Zeng F., Geng K., Liu X., Pan F., He B., Yan W. (2007). Phys. Rev. B: Condens. Matter Mater. Phys..

[cit25] Kayani Z. N., Ishaque R., Zulfiqar B., Riaz S., Naseem S. (2017). Opt. Quantum Electron..

[cit26] MacManus-Driscoll J. L., Khare N., Liu Y., Vickers M. E. (2007). Adv. Mater..

[cit27] Xu X., Blythe H. J., Ziese M., Behan A. J., Neal J. R., Mokhtari A., Ibrahim R. M., Fox A. M., Gehring G. A. (2006). New J. Phys..

[cit28] Behan A. J., Mokhtari A., Blythe H. J., Score D. S., Xu X., Neal J., Fox A. M., Gehring G. A. (2008). Phys. Rev. Lett..

[cit29] Xu Q., Hartmann L., Schmidt H., Hochmuth H., Lorenz M., Schmidt-Grund R., Sturm C., Spemann D., Grundmann M. (2006). Phys. Rev. B: Condens. Matter Mater. Phys..

[cit30] Lu Z.-L., Hsu H.-S., Tzeng Y.-H., Zhang F.-M., Du Y.-W., Huang J.-C. A. (2009). Appl. Phys. Lett..

[cit31] Bellingeri E., Rusponi S., Lehnert A., Brune H., Nolting F., Leveratto A., Plaza A., Marré D. (2019). Sci. Rep..

[cit32] Feng Q., Dizayee W., Li X., Score D. S., Neal J. R., Behan A. J., Mokhtari A., Alshammari M. S., Al-Qahtani M. S., Blythe H. J., Chantrell R. W., Steve M. H., Xu X.-H., Fox A. M., Gehring G. A. (2016). New J. Phys..

[cit33] Ying M., Blythe H. J., Dizayee W., Heald S. M., Gerriu F. M., Fox A. M., Gehring G. A. (2016). Appl. Phys. Lett..

[cit34] Kittilstved K. R., Zhao J., Liu W. K., Bryan J. D., Schwartz D. A., Gamelin D. R. (2006). Appl. Phys. Lett..

[cit35] Neal J., Behan A. J., Ibrahim R. M., Blythe H. J., Ziese M., Fox A. M., Gehring G. A. (2006). Phys. Rev. Lett..

[cit36] Albargi H. B., Alshammari M. S., Museery K. Y., Heald S. M., Jiang F. X., Saeedi A. M., Fox A. M., Gehring G. A. (2019). Coatings.

[cit37] Ravel B., Newville M. (2005). J. Synchrotron Radiat..

[cit38] Troitiño N. F., Viñas S. L., González B. R., Li Z. A., Spasova M., Farle M., Salgueiriño V. (2014). Nano Lett..

[cit39] Score D. S., Alshammari M., Feng Q., Blythe H. J., Fox A. M., Gehring G. A., Quan Z.-Y., Li X.-L., Xu X.-H. (2010). J. Phys.: Conf. Ser..

[cit40] Wang Q., Sun Q., Chen G., Kawazoe Y., Jena P. (2008). Phys. Rev. B: Condens. Matter Mater. Phys..

[cit41] Qi S. F., Jiang F. X., Fan J. P., Wu H., Zhang S. B., Gehring G. A., Zhang Z., Xu X. H. (2011). Phys. Rev. B: Condens. Matter Mater. Phys..

[cit42] Janotti A., Van de Walle C. G. (2007). Phys. Rev. B: Condens. Matter Mater. Phys..

